# Whole-genome resequencing reveals genetic differentiation and selection signatures among wild, local and commercial duck populations

**DOI:** 10.5713/ab.24.0643

**Published:** 2024-11-28

**Authors:** Zhirong Huang, Liyun Zhang, Maojun Luo, Xumeng Zhang, Yunmao Huang, Yunbo Tian, Zhongping Wu, Xiujin Li

**Affiliations:** 1 College of Animal Science & Technology, Zhongkai University of Agriculture and Engineering, Guangzhou, China

**Keywords:** Candidate Gene, Duck, Genetic Diversity, Selection Signal, Whole-genome Sequencing

## Abstract

**Objective:**

The purpose of this study was to systematically analyze the population genetic structure and genetic diversity among wild, local and commercial populations using whole-genome sequencing data from 416 individuals of 22 duck breeds in China and to further explore genetic pathways and candidate genes associated with importantly economic traits.

**Methods:**

We performed principal component analysis, an unrooted neighbor-joining phylogenetic tree and ADMIXTURE to analyze the population structure. We compared the genetic diversity among wild, local and commercial populations using the effective population size, inbreeding coefficient, expected heterozygosity, observed heterozygosity, nucleotide diversity and regions of homozygosity. To detect selection signatures, we calculated the locus-specific branch length for local and commercial populations and calculated genetic differentiation coefficient and genetic diversity between egg and dual-purpose breeds.

**Results:**

Wild, local and commercial duck populations formed three distinct genetic groups. The commercial population presented the lowest genetic diversity, the highest levels of inbreeding and the smallest effective population size. ADMIXTURE analysis also demonstrated that ducks were clearly divided into these three populations at K = 3. Selection signals in the commercial population were associated with growth and muscle development pathways, such as the mTOR signaling pathway and ErbB signaling pathway, and two key genomic regions (Chr1: 70.25 to 74.00 Mb and Chr2: 97.10 to 99.76 Mb) containing important genes, such as *LRP6*, *BORCS5*, and *EDN1*, were identified. In contrast, selection signals in the local population were associated with immune-related pathways involving *NCAM2* and *MPHOSPH6*. Furthermore, *PTGS2* and *PLA2G4A* genes were positively selected in egg breeds, whereas *KCNK16*, *KCNK5*, and *KCNK17* genes were in dual-purpose breeds.

**Conclusion:**

Because of artificial selection, wild, local and commercial populations presented obvious genetic differences. The selection signal analysis revealed that *LRP6*, *BORCS5*, and EDN1 are important for growth and muscle development; *NCAM2* and *MPHOSPH6* are for immune traits; and *PTGS2* and *PLA2G4A* are for egg-related traits.

## INTRODUCTION

Poultry, as an indispensable part of the human food chain, holds an important position in global agricultural production [1]. Ducks, as important poultry, are valuable not only for their meat and egg products but also for their diversity in different cultures and ecosystems [2]. China is one of the largest producers of poultry worldwide and is a rich resource of duck breeds [3]. These duck resources are genetically diverse and exhibit unique adaptability and production performance characteristics.

With the rapid development of molecular technology, whole-genome resequencing has become a powerful tool for studying animal genetic diversity, population structure, selection pressure, and demographic history [4]. Song et al [5] compared Xia’nan cattle with 178 samples from 5 breeds in a public database, which provided a basis for further breeding and improvement of cattle. Gao et al [6] analyzed genomic differences between Chinese indigenous pig breeds and commercial pigs, revealing distinct growth patterns between local and commercial breeds. Jiang et al [7] analyzed the whole-genome sequences of 101 domestic ducks to reveal the complex population history and significant gene flow between duck populations. Li et al [8] conducted a comparative analysis of eight Muscovy duck populations and reported significant differences in fatty acid metabolism and immune-related genes among different breeds. Jiang et al [7] collected genomic data from 78 ducks in China, Southeast Asia and South Asia, revealing the demographic history, gene flow, and domestication patterns of Southeast/South Asian and Chinese duck populations. Zhu et al [9] assembled three chromosome-level genomes and demonstrated the fat-suppressing function of *NR2F2*, which explains the variation in fat deposition between meat-type breeds. However, these studies are limited by the number of samples and breeds. To date, no study has carried out broader genetic comparative analyses among wild, local, and commercial populations including as many Chinese duck breeds as possible.

In this study, we collected 416 high-quality whole-genome resequencing datasets from 22 breeds, including wild, local, and commercial duck breeds, to represent a wide spectrum of genetic diversity in ducks in China. The objective of this study was to analyze the whole-genome data of these duck breeds comprehensively to reveal their genetic diversity, population genetic structure, selection signals, and candidate genes related to economically important traits. This study provides a scientific basis for Chinese duck protection and genetic improvement.

## MATERIALS AND METHODS

### Whole-genome resequencing data analysis

In this study, we downloaded publicly available whole-genome resequencing data of 484 Chinese duck individuals from the National Center for Biotechnology Information Sequence Read Archive database prior to November 26, 2023. We adopted a stringent and uniform pipeline to analyze raw genome data and filter single nucleotide polymorphism (SNP)s. Briefly, we first trimmed adaptors and removed low-quality reads via Trimmomatic (v0.39) with the following parameters: LEADING: 3 TRAILING: 3 SLIDINGWINDOW: 4:15 MINLEN: 36. We then mapped the clean reads to the Pekin duck (Anas platyrhynchos) reference genome (ZJU1.0) via the Burrows-Wheeler Aligner (v0.7.17) [10] with the parameters mem -M–R. We used SAMtools (1.13) [11] to sort bam files and the MarkDuplicates module in GATK (4.2.3.0) [12] to remove duplicates. We used mosdepth (0.3.3) [13] to calculate the sequencing depth of each sample and selected samples with sequencing depths greater than 3×. Finally, we called variants through the modules HaplotypeCaller, CombineGVCF, and GenotypeGVCF in GATK (4.2.3.0) and filtered SNPs via the module SelectVariants with the following parameters: QD<2.0 || MQ<40.0 || FS>60.0 || SOR>3.0 || MQRankSum<−12.5 || ReadPosRankSum<−8.0. We utilized BCFtools (v1.9) [11] with the parameters --min-af 0.01:minor -e ‘F_MISSING>0.9’ -m 2 -M 2 -v snps to obtain biallelic SNPs, and these SNPs were then more rigorously filtered via PLINK (v1.9) [14] with the parameters minor allele frequency<0.05 and Hardy-Weinberg equilibrium <0.000001.

After removing 68 samples with sequencing depths below 3×, we filtered 416 high-quality individual ducks with an average depth of 7× coverage for the following analysis. These samples were derived from Bioproject IDs: PRJNA599025, PRJNA658213, PRJNA888455, PRJNA409830, PRJNA419832, PRJNA450892, and PRJNA549423. We categorized these 416 ducks into wild populations (72), local populations (159), and commercial populations (185) (Table 1). The wild breed included 72 Mallard ducks, whereas the commercial populations included Pekin ducks, Mapleleaf ducks and Cherry Valley ducks. The local populations included 18 breeds, i.e., JinDing duck, Liancheng White duck, MaWang duck, Putian Black duck, SanSui duck, ShanMa duck, ShaoXing duck, Taiwan duck, Yulin-Wu duck, DongLan duck, YouXian duck, GaoYou duck, Ji’an Red duck, Longsheng-Cui duck, WenQiao duck, Rongshui-Xiang duck, Xilin-Ma duck, and Yulin-Ma duck.

### Annotation of genetic variants

After quality control, we obtained 5,140,908 high-quality SNPs, and these SNPs were annotated via the ANNOVAR package [15] on the basis of the gene annotations of the Pekin duck reference genome (ZJU1.0). We divided these SNPs into five classes: exon regions, intron regions, splicing sites, upstream and downstream regions and intergenic regions.

### Population genetics analysis

We performed principal component analysis (PCA) for 416 ducks by using PLINK (v1.9), with 5,140,908 SNPs shared among the three populations. We constructed an unrooted neighbor-joining (NJ) phylogenetic tree via iTOL [16] on the basis of the identity-by-state genetic distance matrix among 416 individuals calculated via PLINK (v1.9). Population genetic structure was inferred via ADMIXTURE software (v-1.3.0) [17], which employs a maximum likelihood-based method to estimate individual ancestries from multilocus SNP genotype datasets. We then performed visualization by using TBtools-II [18] while setting a predefined range of genetic clusters (K) from 2 to 12 to encompass the maximum number of lineages.

We estimated the population genetic diversity of the wild, local and commercial populations. The genetic diversity included the effective population size (Ne), inbreeding coefficient (F), expected heterozygosity (He), observed heterozygosity (Ho), nucleotide diversity (Pi), and regions of homozygosity (ROH). The effective population size (Ne) of different populations was estimated via SNeP (v1.1) [19] with independent SNPs calculated via PLINK (v1.9) with the parameters --indep-pairwise 50 5 0.2, and 72 samples from each population were used to avoid the effect of the number of samples. We selected 72 individuals from commercial and local populations on the basis of the corresponding proportion of each breed included in each population. We calculated F (--het), He (--hardy), Ho (--hardy) and ROH (--homozyg --homozyg-window-snp 50 --homozyg-window-het 1 --homozyg-snp 50 --homozyg-window-missing 5 --homozyg-window-threshold 0.05 --homozyg-density 500 --homozyg-gap 1000) for each duck of the wild population, local population, and commercial population, respectively. We calculated F, He, Ho, and ROH for each duck from the wild population, local population, and commercial population, respectively. We used VCFtools (v1.17) [12] to compute Pi values by scanning all genomic regions within 50-kb sliding windows with a step size of 25 kb. We compared the patterns of linkage disequilibrium (LD) among wild ducks, local ducks, and commercial ducks through pairwise LD estimates, which were measured as r^2^ values calculated from PopLDdecay (v3.42) [20]. The samples used for the LD estimates were the same as those used for the Ne calculation.

### Selective signatures and functional enrichment

First, we calculated genetic differentiation coefficient (Fst) values for the pairwise populations among the wild population, local population and commercial population using VCFtools (v1.17) with 50-kb sliding windows and a step size of 25 kb. To identify selective genomic signals in commercial or local populations compared with those in the other two populations, we then derived the locus-specific branch length (LSBL) [21] from the pairwise Fst distances calculated as follows: LSBL_CM–(LC+WT)_ = (Fst_CM–LC_ + Fst_CM–WT_ − Fst_LC–WT_)/2 and LSBL_LC–(CM+WT)_ = (Fst_CM–LC_ + Fst_LC–WT_ − Fst_CM–WT_)/2, where Fst_CM–LC_ refers to the Fst value between the commercial population and the local population, Fst_CM–WT_ refers to the Fst value between the commercial population and the wild population, and Fst_LC–WT_ refers to the Fst value between the local population and the wild population. We selected the top 1% of LSBL values of the genomic windows as candidate regions. Moreover, for the local population, we also analyzed selective signatures between egg and dual-purpose breeds by calculating Fst and Pi values from VCFtools (v1.17) with 50-kb sliding windows and a step size of 25 kb. We annotated the genes related to these important genomic regions, and we utilized the clusterProfiler R package [22] for the Gene Ontology (GO) and Kyoto Encyclopedia of Genes and Genomes (KEGG) pathway analyses of these candidate genes. The GO and KEGG pathways were selected from the top 10 enriched pathways in terms of Benjamini-adjusted p values. For some importantly selected genomic regions, we employed LSBL and Pi values with a 10-kb sliding window and a 5-kb step size across wild, local, and commercial populations to conduct comparisons and to identify candidate genes within these selected genomic regions.

## RESULTS

### Whole-genome sequencing and single nucleotide polymorphism calling

**Figure 1:**
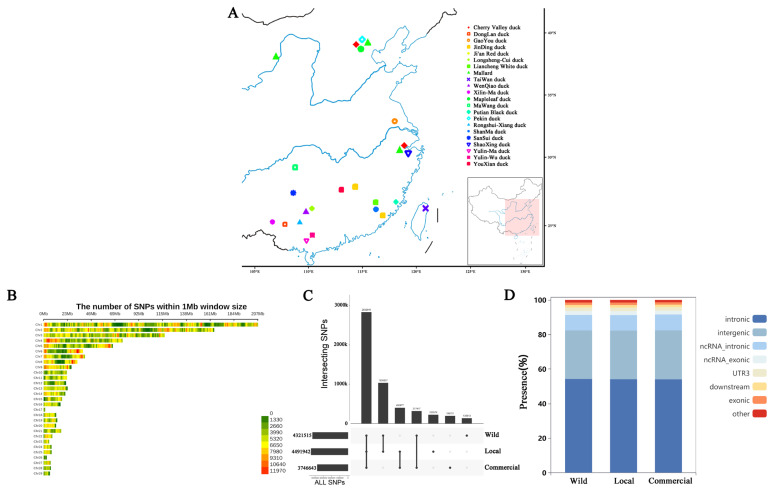
Geographic distributions and genetic variants of three duck populations. (A) Geographical distribution of 22 duck breeds. (B) The distribution of SNPs across 29 chromosomes. (C) The shared and unique SNPs among the wild, local, and commercial duck populations. (D) Functional annotation of SNPs for wild, local, and commercial duck populations. SNP, single nucleotide polymorphism.

We obtained 416 ducks with a wide and representative geographic distribution across various regions in China, yielding 5,140,908 high-quality SNPs distributed across 29 chromosomes (Figures 1A, 1B). As shown in Figure 1C, among these SNPs, we identified 4,321,515 SNPs in the wild population, 4,491,942 in the local population, and 3,746,643 in the commercial population. There were 2,832,649 SNPs shared among these three populations, accounting for approximately 55.1% of the total SNPs. The commercial population had 195,701 unique SNPs, the local population had 222,579 unique SNPs, and the wild population had 135,613 unique SNPs.

In the functional annotation of SNPs, the percentages of SNPs located in introns, intergenic regions, introns and exons of noncoding RNAs, untranslated regions3 regions, downstream, and exonic regions were very similar among the wild, local, and commercial populations. For example, the majority of SNPs are within introns, accounting for approximately 54.2%, 54.06%, and 54% of the total number of SNPs in the wild, local, and commercial populations, respectively. In contrast, the proportions of SNPs located in exons were relatively low, i.e., approximately 1.4% for these three populations (Figure 1D).

### Population genomic analysis

**Figure 2:**
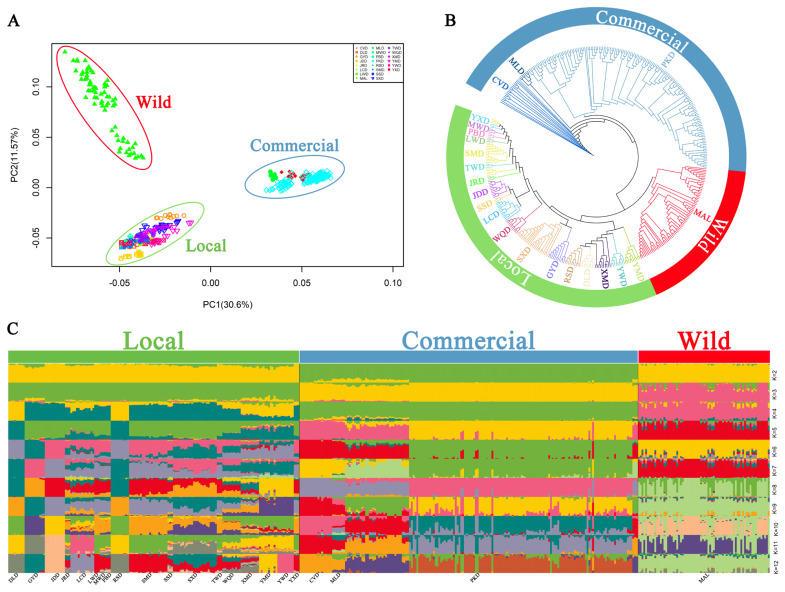
Population structure of 22 duck breeds based on 5,140,908 common single nucleotide polymorphisms. (A) Principal component analysis (PCA) clustering of 416 samples. (B) Identity-by-state (IBS) neighbor joining (NJ) tree constructed with 416 samples. The wild population encompasses mallards (MALs). The local population is represented by various breeds, including JinDing duck (JRD), Liancheng White duck (LCW), MaWang duck (MWD), Putian Black duck (PBD), SanSui duck (SSD), ShanMa duck (SMD), ShaoXing duck (SXD), Taiwan duck (TWD), Yulin-Wu duck (YWD), DongLan duck (DLD), YouXian duck (YXD), GaoYou duck (GYD), Ji’an red duck (JRD), Longsheng-Cui duck (LCD), WenQiao duck (WQD), Rongshui-Xiang duck (RSD), Xilin-Ma duck (XMD), and Yulin-Ma duck (YMD). Commercial populations include Pekin ducks (PKD), Mapleleaf ducks (MLD) and Cherry Valley ducks (CVDs). (C) Model-based clustering of 416 samples calculated via ADMIXTURE with K values ranging from 2 to 12.

PCA clearly revealed that individuals from the wild, local, and commercial populations were clustered, with PC1 and PC2 accounting for 30.61% and 11.57% of the total variation, respectively (Figure 2A). The NJ tree also confirmed that the ducks were divided into three groups: wild, local, and commercial populations (Figure 2B). Moreover, ADMIXTURE analysis indicated that the wild, local, and commercial populations were clearly distinguishable at K = 3, with a log-likelihood value of −1,637,271,699.91 and a cross-validation error of 0.49702 (Figure 2C).

**Figure 3:**
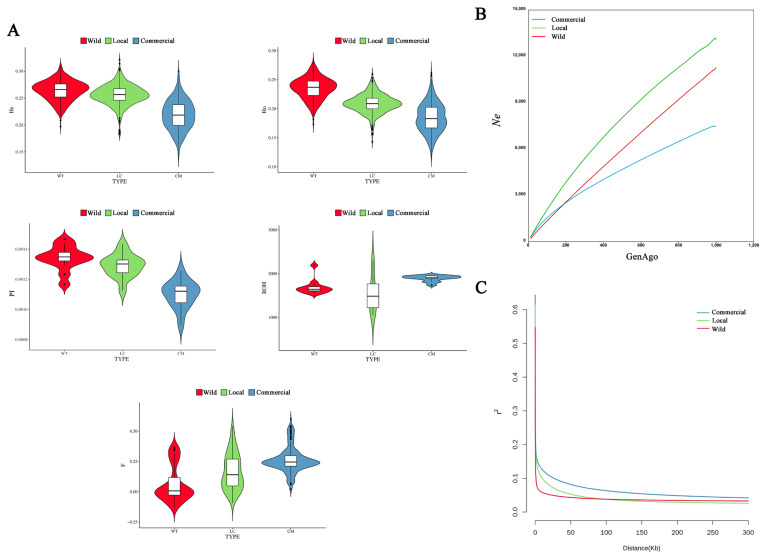
Genetic diversity of the three duck populations. (A) Expected heterozygosity (He), observed heterozygosity (Ho), nucleotide diversity (Pi), length of regions of homozygosity (bp), and inbreeding coefficient (F) values of three duck populations. (B) Effective population size for three populations in different generations. (C) Estimation of the genome-wide linkage disequilibrium (LD) decay for three duck populations.

As shown in Figure 3A, in terms of Ho, He, and Pi, commercial duck breeds presented the lowest values (He = 0.22±0.15, Ho = 0.19±0.13, and Pi = 0.0011±0.0009), suggesting a relatively high degree of inbreeding. This was supported by the inbreeding coefficient, where commercial breeds also had the highest value (F = 0.26±0.15). The commercial duck breeds also presented the longest average ROH length (1905.33±1371.00 kb) among the three populations. The commercial population had the smallest Ne at generation 999, except that the Ne of the wild population was lower than that of the commercial population before generation 150, while the local population consistently had the largest Ne across all generations (Figure 3B). Compared with the other two populations, the commercial population presented a greater extent of LD (r^2^) (Figure 3C). These results were consistent with our general understanding of the wild, local, and commercial populations.

### Genomic signatures

**Figure 4:**
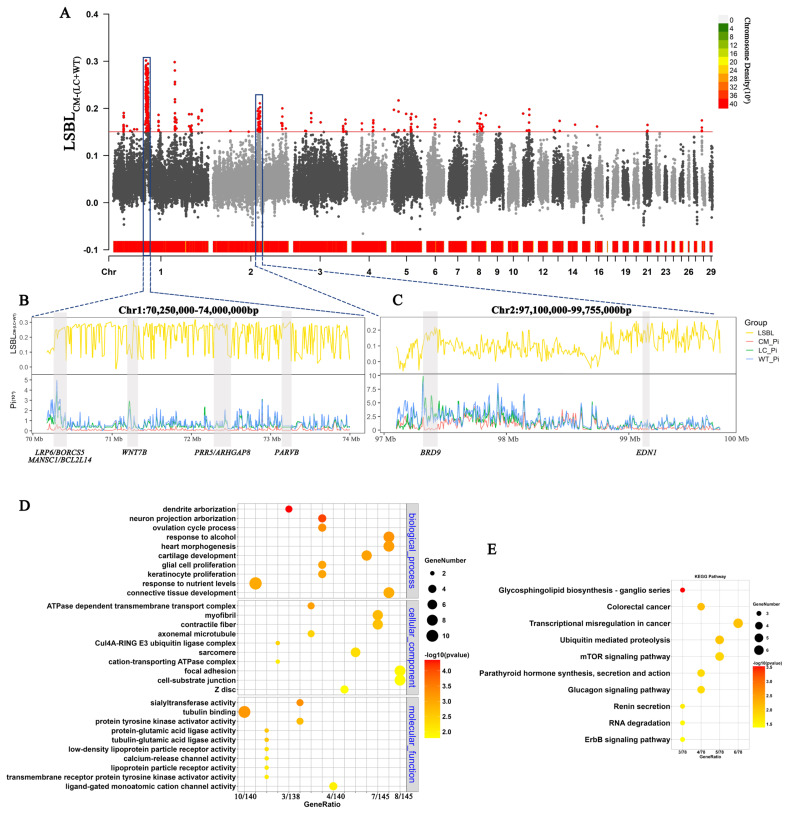
Genome-wide selective signals of commercial populations compared with those of wild and local populations. (A) Manhattan plots of LSBL_CM–(LC+WT)_ values between commercial and the other two populations. (B) LSBL_CM–(LC+WT)_ and Pi with a 10-kb sliding window and 5-kb step and candidate genes in the Chr1: 70.25–74.00 Mb region. (C) LSBL and Pi with the 10-kb sliding window and 5-kb step and candidate genes in the region in the region Chr2: 97.10–99.76 Mb. (D) Gene Ontology enrichment analysis of genes located in the top 1% of the LSBL_CM–(LC+WT)_. (E) Kyoto Encyclopedia of Genes and Genomes enrichment analysis of genes located in the top 1% of the LSBL_CM–(LC+WT)_. LSBL, locus-specific branch length; Pi, nucleotide diversity.

As shown in Figure 4A, we identified 152 genes by using the top 1% LSBL_CM–(LC+WT)_ genome regions of the commercial duck breeds. We subsequently conducted KEGG and GO enrichment analyses for these genes (Figures 4D, 4E). For the KEGG pathways, the mTOR signaling pathway and ErbB signaling pathway were significantly enriched (p<0.05). The GO enrichment analysis revealed that the enriched pathways were highly related to muscle development, such as cartilage development, connective tissue development, myofibrils and contractile fibers. Furthermore, we analyzed two important genomic regions by calculating LSBL and Pi with a 10-kb sliding window and 5-kb step and identified the candidate genes *LRP6, BORCS5, MANSCI, BCL2L14, WNT7B*, and *PRR5* in the region Chr1: 70.25 to 74.00 Mb (Figure 4B) and *BRD9* and *EDN1* in the region Chr2: 97.10 to 99.76 Mb (Figure 4C).

**Figure 5:**
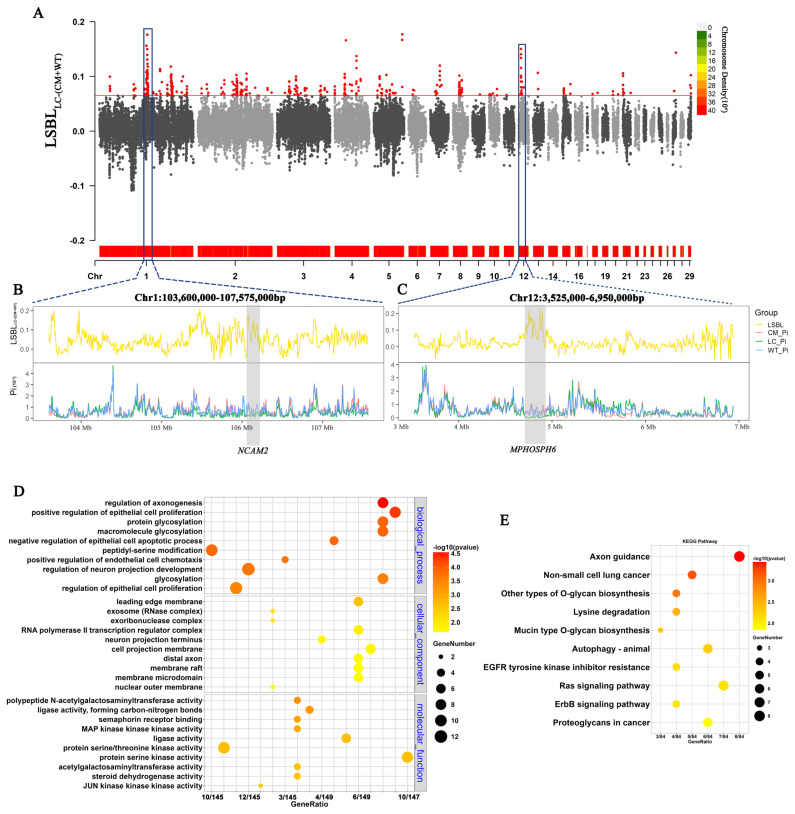
Genome-wide selective signals of local populations compared with those of wild and commercial populations. (A) Manhattan plots of LSBL values between the local population and the other two populations. (B) LSBL_LC–(CM+WT)_ and Pi with a 10-kb sliding window and 5-kb step and candidate genes in the region Chr1: 103.6–107.57 Mb. (C) LSBL_LC–(CM+WT)_ and Pi with the 10-kb sliding window and 5-kb step and candidate genes in the region Chr12: 3.52–6.95 Mb. (D) Gene Ontology enrichment analysis of genes located in the top 1% of the LSBL_LC–(CM+WT)_. (E) Kyoto Encyclopedia of Genes and Genomes enrichment analysis of genes located in the top 1% of LSBL_LC–(CM+WT)_. LSBL, locus-specific branch length; Pi, nucleotide diversity.

Similarly, we detected 277 genes involved by using the top 1% of LSBL_LC–(CM+WT)_ values (Figure 5A). Through KEGG enrichment analysis, other types of O-glycan biosynthesis and mucin type O-glycan biosynthesis were significantly enriched (p<0.05), which may be linked to the immune system and disease resistance of the local population (Figure 5E). The significantly enriched GO pathways were regulation of axonogenesis, regulation of neuron projection development, protein glycosylation, and macromolecule glycosylation (Figure 5D). These pathways have implications for neurodevelopment and protein glycosylation processes. By using LSBL and Pi values with a 10-kb sliding window and 5-kb step for two important genomic regions, we identified the candidate gene *NCAM2* in the region Chr1: 103.60 to 107.58 Mb (Figure 5B) and *MPHOSPH6* in the region Chr12: 3.53 to 6.95 Mb (Figure 5C).

**Figure 6:**
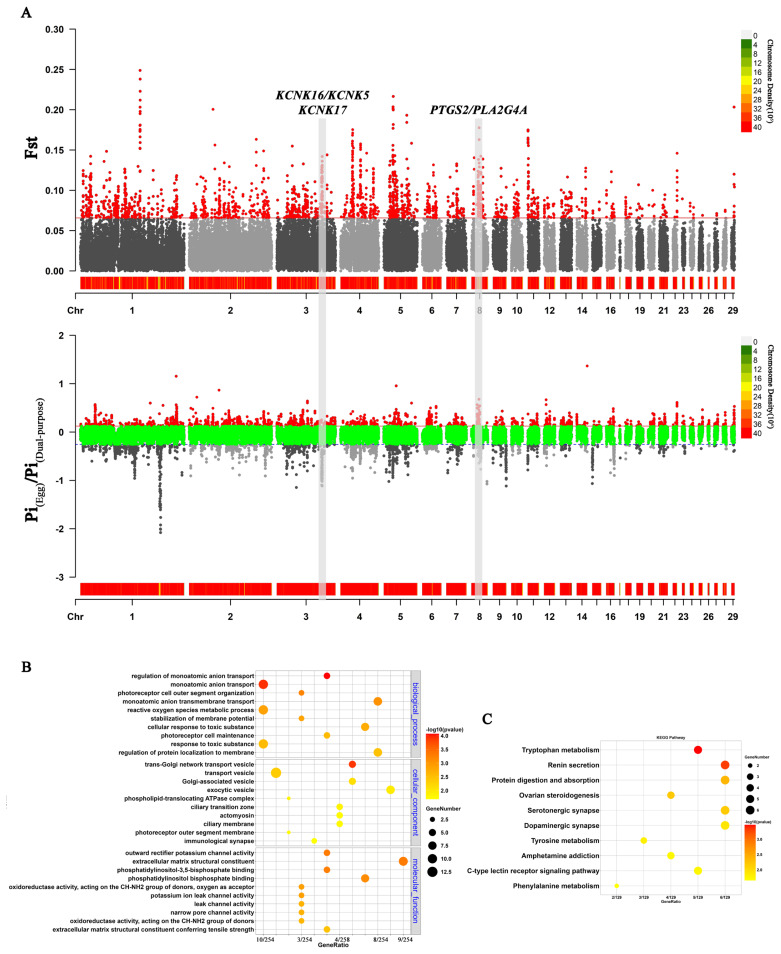
Genome-wide selective signals of the egg-laying population compared with those of dual-purpose populations. (A) Manhattan plots of Fst and Pi ratio values between egg-laying and dual-purpose populations. (B) Gene Ontology enrichment analysis of genes located in the top 5% of the Fst genes. (C) Kyoto Encyclopedia of Genes and Genomes enrichment analysis of genes located in the top 5% of the Fst genes. Fst, genetic differentiation coefficient; Pi, nucleotide diversity.

We identified 415 genes in the overlap of the top 5% Fst and Pi ratios between egg-laying and dual-purpose ducks in the local population. For these genes, KEGG pathway analysis revealed that the ovarian steroidogenesis pathway and protein digestion and absorption pathway were significantly enriched (p<0.05) (Figure 6C). GO analysis revealed that the regulation of monoatomic anion transport were the most significantly enriched terms (Figure 6B). We identified two candidate genes, i.e., *PTGS2* and *PLA2G4A*, which are involved in the ovarian steroidogenesis pathway, that are positively selected in the egg laying population, whereas *KCNK16*, *KCNK5* and *KCNK17* are positively selected in dual-purpose ducks and are involved in the fundamental processes of protein digestion and absorption and monoatomic anion transport.

## DISCUSSION

In this study, we conducted a preliminary analysis of the most comprehensive duck dataset of whole-genome resequencing data from 416 wild, local, and commercial individuals in China. The three populations presented significant differences in genetic diversity and in the distribution of SNPs. The local population has the highest numbers of total and unique SNPs, indicating greater genetic diversity. The wild population has slightly fewer SNPs but still maintains high diversity, whereas the commercial population has the fewest SNPs and a greater proportion of unique SNPs, suggesting that it has been subjected to stronger artificial selection pressure. The trends of Pi, F, He, and Ho across wild, local and commercial populations indicated that the wild population presented relatively high genomic diversity, which was confirmed in previous whole-genome resequencing analyses [23]. The analysis of ROH and LD decay also revealed the rich genetic diversity and the presence of inbreeding in the wild population. The Ne of the local population was the highest among the three populations, largely because it included 18 breeds. Commercial populations presented lower genetic diversity and higher inbreeding coefficients, which are likely associated with strong artificial selection and breeding strategies [24]. Local populations exhibit relatively high genetic diversity, possibly due to their long-term adaptation to specific environmental conditions and limited human intervention [25]. These differences reflect the distinct evolutionary trajectories of each population in terms of breeding and environmental adaptation.

In the commercial population, we detected selection signals for genes associated with cell growth, proliferation, and differentiation, such as those in the mTOR and ErbB signaling pathways. These signals confirmed that commercial populations have been artificially selected for growth rate and meat quality [26,27]. It has been reported that *LRP6* is involved in the bone construction process [28] and that *BRD9* has a positive effect on the development of muscles and bones [29]. *ARHGAP8* is reported for the first time in this study. Additionally, *EDN1*, *WNT7B*, *PARVB*, and *PRR5* are related to the development of muscle and adipose tissue. *EDN1* has a significant effect on the development of meat marbling [30]. *WNT7B* promotes bone formation and muscle development [31]. *PARVB* establishes and maintains cell polarity and the actin cytoskeleton, affecting muscle development [32]. *PRR5* is associated with fat deposition in pigs [33].

In the local population, we observed selection signals related to the immune system and disease resistance, such as the O-glycosylation pathway [34]. This may reflect the adaptation of local duck breeds to specific environmental pressures, such as challenges from pathogens and parasites. Selection signals related to neural development and glycosylation processes may influence the environmental adaptability and reproductive capacity of local duck breeds [35,36]. *NCAM2* promotes neural remodeling and synaptic plasticity, supporting adaptation to rapid environmental changes and stress [37]. Polymorphisms in the *MPHOSPH6* gene are associated with an increased risk of several diseases, suggesting that it may play a key role in immune regulation and inflammatory responses [38–40].

To identify selection signals between egg-laying and dual-purpose ducks in the local population, we identified signaling pathways associated with reproduction and egg production, such as ovarian steroid production. Interestingly, we found that *PTGS2* and *PLA2G4A*, which are highly expressed in egg-laying ducks, are involved in ovarian steroid production and may play important roles in egg production. *PTGS2* on Chr08: 17.77 to 17.95 Mb plays a key role in prostaglandin synthesis and exerts an important impact on ovarian function and ovulation. *PLA2G4A* indirectly regulates prostaglandin synthesis, thereby affecting egg production and egg production performance. These two genes were reported to influence egg production performance in chickens [41]. We also detected *KCNK16*, *KCNK5*, and *KCNK17* at Chr03: 89.45 to 90.18 Mb, which are positively selected in dual-purpose ducks. According to the results of the GO enrichment analysis, these genes are involved in monoatomic anion transport and protein digestion and are critical for maintaining acid-base balance, respiratory function, and the stability of the intracellular environment.

## CONCLUSION

To date, we have conducted the most comprehensive preliminary analysis of whole-genome resequencing data from 416 wild, local, and commercial ducks in China. Owing to strong artificial selection, the commercial population has the lowest genetic diversity and the highest inbreeding level. The selection signals in the commercial population are related to growth and muscle development, with *LRP6*, *BORCS5*, *MANSCI*, *BCL2L14*, *WNT7B*, *PRR5*, *BRD9*, and *EDN1* likely playing key roles. In contrast, selection signals in the local population were associated with immune-related pathways involving *NCAM2* and *MPHOSPH6*. Furthermore, the *PTGS2* and *PLA2G4A* genes were positively selected in egg breeds, whereas the *KCNK16*, *KCNK5*, and *KCNK17* genes were positively selected in dual-purpose breeds. This study provides valuable insights into the genetic mechanisms of local and commercial populations, supporting the development of strategies for conserving genetic diversity and optimizing breeding management.

## Figures and Tables

**Table 1:** Sample information for 416 ducks used in this study

Population	Breed	Count	Type	Location	Sequencing depth (×)
Wild	Mallard	72	Wild	Zhejiang/Ningxia	7.54
Local	JinDing duck	11	Egg	Longhai, Fujian	6.11
	Liancheng White duck	3	Egg	Liancheng, Fujian	9.74
	Mawang duck	3	Egg	Mawang, Chongqing	9.05
	Putian black duck	3	Egg	Putian, Fujian	9.78
	Sansui duck	3	Egg	Sansui, Guizhou	8.98
	Shanma duck	21	Egg	Longyan, Fujian	5.47
	Shaoxing duck	24	Egg	Shaoxing, Zhejiang	4.75
	Taiwan duck	3	Egg	Taiwan	9.25
	Youxian duck	3	Egg	You, Hunan	10.19
	Yulin-Wu duck	9	Dual-purpose	Yulin, Guangxi	8.64
	Donglan duck	9	Dual-purpose	Donglan, Guangxi	8.52
	Gaoyou duck	11	Dual-purpose	Gaoyou, Jiangsu	6.24
	Ji’an red duck	3	Dual-purpose	Ji’an, Jiangxi	9.90
	Longsheng-Cui duck	13	Dual-purpose	Longsheng, Guangxi	8.86
	Wenqiao duck	10	Dual-purpose	Wenqiao, Guangxi	9.27
	Rongshui-Xiang duck	10	Dual-purpose	Rongshui, Guangxi	9.66
	Xilin-Ma duck	10	Dual-purpose	Xilin, Guangxi	8.87
	Yulin-Ma duck	10	Dual-purpose	Yulin, Guangxi	8.85
Commercial	Pekin duck	160	Meat	Beijing	6.58
	Mapleleaf duck	7	Meat	Beijing	4.93
	CherryValley duck	18	Meat	Beijing/Zhejiang	4.57
